# Climate change risk to forests in China associated with warming

**DOI:** 10.1038/s41598-017-18798-6

**Published:** 2018-01-11

**Authors:** Yunhe Yin, Danyang Ma, Shaohong Wu

**Affiliations:** 1 Key Laboratory of Land Surface Pattern and Simulation, Institute of Geographic Sciences and Natural Resources Research, Chinese Academy of Sciences, 11A, Datun Road, Chaoyang District, Beijing 100101 China; 2 University of Chinese Academy of Sciences, 19A Yuquan Road, Shijingshan District, Beijing 100049 China

## Abstract

Variations in forest net primary productivity (NPP) reflects the combined effects of key climate variables on ecosystem structure and function, especially on the carbon cycle. We performed risk analysis indicated by the magnitude of future negative anomalies in NPP in comparison with the natural interannual variability to investigate the impact of future climatic projections on forests in China. Results from the multi-model ensemble showed that climate change risk of decreases in forest NPP would be more significant in higher emission scenario in China. Under relatively low emission scenarios, the total area of risk was predicted to decline, while for RCP8.5, it was predicted to first decrease and then increase after the middle of 21st century. The rapid temperature increases predicted under the RCP8.5 scenario would be probably unfavorable for forest vegetation growth in the long term. High-level risk area was likely to increase except RCP2.6. The percentage area at high risk was predicted to increase from 5.39% (2021–2050) to 27.62% (2071–2099) under RCP8.5. Climate change risk to forests was mostly concentrated in southern subtropical and tropical regions, generally significant under high emission scenario of RCP8.5, which was mainly attributed to the intensified dryness in south China.

## Introduction

Climate change has been suggested as a major driver of the dynamics of terrestrial ecosystems through its influence on vegetation growth and distribution^1–4^. Ecosystems may be at risk if their resilience and ability to adapt are severely damaged. An increase in global warming will result in greater risks for ecosystems over the 21st century^5,6^. For example, if the temperature increases by more than 3 °C, 44% of global terrestrial ecosystems risk conversion from carbon sinks to carbon sources^5^. Forest, the most complicated of all terrestrial ecosystems^7^, plays an important role in the carbon cycle and accounts for 49% of terrestrial gross primary production^8^. The annual gross carbon uptake by global forests equates to roughly half of the carbon emitted from fossil fuels^9^. Research into the spatial and temporal patterns of the climate change risk faced by forests is crucial to determine priority areas that should be targeted for action to manage the consequences of climate change.

Because of higher temperatures and incidental droughts, tree mortality and related dieback may increase, posing threats to carbon storage, biodiversity, and production in forests^10–12^. Net primary productivity (NPP) refers to the net amount of carbon fixed by plants through photosynthesis per unit time and area, namely the difference between gross primary productivity and autotrophic respiration^13,14^. Because the loss of NPP is considered to be unfavorable for terrestrial carbon sinks and ecosystem functioning, it has been used to indicate the risks of climate change for ecosystems^6,15,16^. In general, NPP is an indicator of plant growth and reflects the capacity of vegetation to sequester and convert the products of photosynthesis^17–19^. Changes in the NPP of terrestrial ecosystems could effectively reflect the substantial spatial and temporal heterogeneity in climatic, ecological, geochemical, and human influences on the biosphere^1,20–22^.

The impacts of climate change on forest NPP are complex. Warming and prolongation of the growing season may enhance forest NPP in high latitude and alpine areas^23–25^. In contrast, other factors such as drought, heat waves, wildfire, and insect disturbances may cause extensive reductions in NPP in some forests^26–31^. Of these factors, drought or dryness have often resulted from increases in the atmospheric evaporative demand (AED) and soil water deficit, and from decreases in precipitation. However, there is uncertainty about whether the current trends of regional aridity will intensify or weaken, which partly reflect AED estimation methods^32–37^. Generally, there is risk if the reduction in NPP caused by the overall effects of climate change indicates possible damage or corruption, such as poor vegetation coverage, the expansion of desertification^38^ and the decline of ecosystem services ability^39^, other than the lack of vegetation productivity and biomass. For example, based on version 2 of the Atmosphere Vegetation Interaction Model and the IPCC SRES B2 scenario, Shi *et al*.^40^ recently assessed the risks to ecosystems mainly including forest, shrubland, grassland and cultivated land in China from climate change when (1) the adverse variance was beyond the typical natural variability of NPP, and (2) there was the possibility that, in the future, the NPP would fall below the minimum NPP during the baseline period. However, we have little information regarding how the risks might respond to climate change, though it is important when assessing future impacts in forests in China. More importantly, there have been few quantitative assessments of the rates at which risk might change as regional mean temperatures increase.

The primary objectives of this study were therefore to (1) quantify the spatio-temporal patterns of different levels of risks indicated by the loss of forest NPP under future climate change in China, (2) investigate how the rate of regional climate change risks would vary in response to warming in the future, and (3) address the relative contributions of climatic factors to the risks. To achieve these objectives, we modeled NPP by modifying the AED sub-model in the Lund–Potsdam–Jena Dynamic Global Vegetation Model (LPJ-DGVM), which was driven by a set of general circulation models (GCMs) with representative concentration pathways (RCPs) that covered the period 1981–2100. Our research highlighted the different levels of risks to forests from climate change, and it should give us an improved understanding of how forest ecosystems will respond to future climate change.

## Materials and Methods

### Study area

Forests in China cover about 21.63% (2009–2013) of the land surface and have a carbon stock of approximately 84.27 billion tons. They account for a significant component of terrestrial ecosystems in China and represent the largest afforested area in the world^41^. Hou^42^ reported that needleleaf forest, the most widespread forest vegetation type, extended from the cold temperate zone to the tropical zone, and was mainly concentrated in southwestern and northeastern China. This forest type is dominated by *Pinaceae*, *Taxodiaceae*, and *Cupressaceae*. Mixed needleleaf and broadleaf forest is mainly found in northeastern China (e.g. *Pinus koraiensis*) and mountainous areas of southern China (e.g. *Tsuga* spp. and *Chamaecyparis*). Broadleaf forest is mainly concentrated in the eastern part of Northeast China, south of the Qinling Mountains, and in the southeastern part of the Tibetan Plateau.

**Figure 1:**
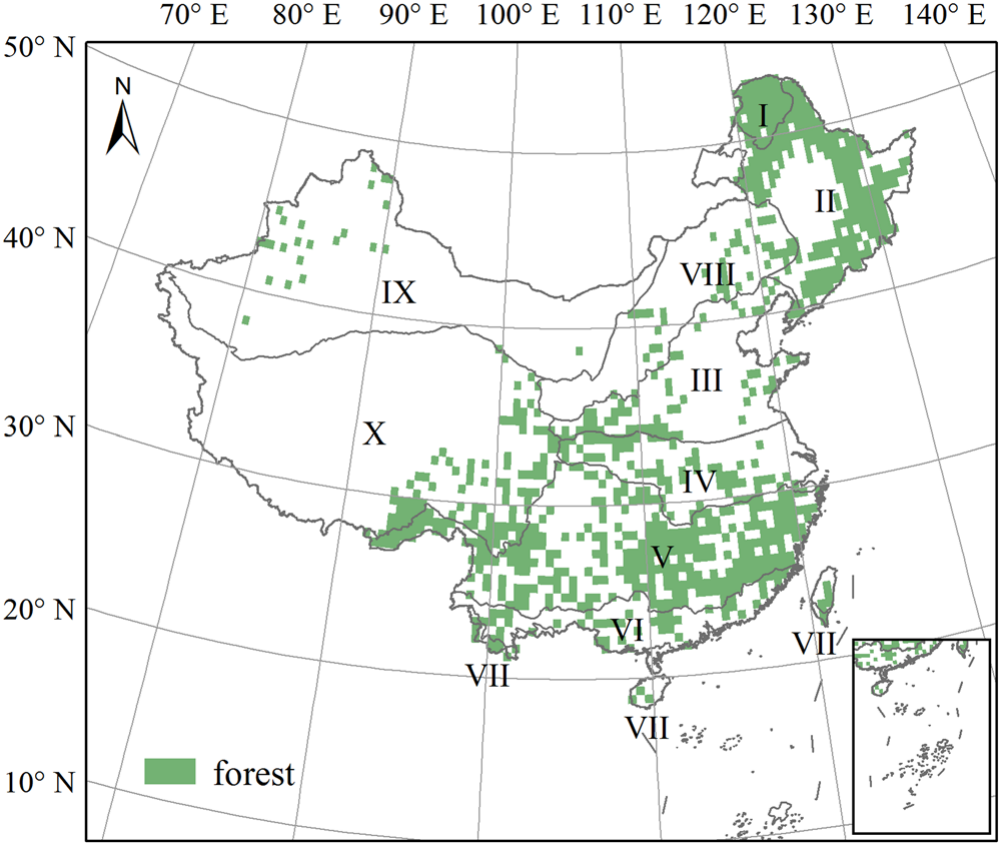
Forest distribution and eco-geographical regions in China. I: Cold temperate humid region; II: Mid-temperate humid/sub-humid region; III: Warm temperate humid/sub-humid region; IV, Northern subtropical humid region; V: Mid-subtropical humid region; VI: Southern subtropical humid region; VII: Tropical humid region; VIII: North semi-arid region; IX: Northwest arid region, and X: Tibetan Plateau region. The forest distribution data were obtained from the 1:1000000 vegetation map of China^42^. The eco-geographical regions boundary was adopted from Zheng^43^. The figure was generated using ArcGIS 10.1 software (http://www.esri.com/).

We chose our study area from the distribution of forest types presented on the 1:1,000,000 vegetation map of China^42^ and the distribution of eco-geographical regions in China^43^, as shown in Fig. 1. We simulated NPP from 1981 to 2099 at a spatial resolution of 0.5° × 0.5° with the modified LPJ-DGVM.

### Risk assessment

Changes in ecosystem state are generally described by the anomaly of future projections from the historical long-term average, with the assumption that the standard deviation of the state variable represents the ecosystem natural interannual variability (IAV)^5,16,44,45^. NPP is defined as the rate of accumulation of carbon after losses from plant respiration and other metabolic processes that maintain the plant’s living systems are taken into account^46^. In this study, we considered that there was risk when the absolute value of future negative anomaly of NPP exceeded the IAV for the period from 1981 to 2010, based on the LPJ simulations. The risks were ranked to correspond with the multiple relationships between the decreases in the NPP and the baseline IAV. Using the simulated NPP, we judged the risk at each pixel and counted the area of forest at risk based on smoothed data, i.e. 30-year running mean NPP. Linear trends in the time series of risk were detected by the Ordinary Least Squares (OLS) method; the statistical significance of the trends was determined by the non-parametric Mann-Kendall test.

Specifically, we calculated the anomalies of the forest NPP in China for future periods (from 2021 to 2050 and from 2071 to 2099) from the baseline period (from 1981 to 2010). Positive anomalies indicated no risk. Negative anomalies were compared with their baseline standard deviation to indicate different levels of risk for each pixel. In line with the criteria and indicators applied by Scholze *et al*.^5^ and Heyder *et al*.^16^, the risks were deemed low, medium, and high level when the absolute values of the negative NPP anomaly were less than half of the standard deviation (*α* < 0.5), greater than half but less than one standard deviation (0.5 < *α* < 1), or greater than one standard deviation (*α* > 1), respectively. In this study, we paid more attention to abnormal variabilities in the NPP that were less than the average minus a number of standard deviations, as a certain loss of productivity outside the typical natural NPP variability was perceived to be an unacceptable impact from climate change^47^. From this, we then classified the risk levels from the multiple relationships between the anomalies and standard deviation.

The standard deviation was computed as follows:

1 $$\delta =\sqrt{\frac{\sum _{i=1}^{n}{({x}_{i}-\bar{x})}^{2}}{n-1}}$$

where *δ* was the standard deviation at the pixel scale, ${x}_{i}$ was the annual NPP of year *i*, $\bar{x}$ was the average NPP of the baseline period, and *n* was the total number of baseline years (*n* = 30).

The multiple *α* was determined by the following equation:

2 $$\alpha =\frac{|{x}_{i}-\overline{x|}}{\delta }$$

The risk levels of forest productivity were classified from the above multiples.

### LPJ Model

Forest NPP in China was projected with the process-based LPJ model^48^. In the LPJ model, NPP is calculated by subtracting maintenance and growth respiration from GPP, which is computed by coupling the photosynthesis and water balance schemes. The formula is as follows:

3 $${\rm{NPP}}=0.75({\rm{GPP}}-{R}_{m})$$

where R_m_ is the maintenance respiration and 0.75 is the ratio of NPP in the remainder, considering 0.25 is the growth respiration coefficient.

Proven as a useful tool for simulating the structure and function of large-scale ecosystems, the LPJ is driven by data of monthly climate and soil texture. The model comprises 10 plant functional types (PFTs), defined by bioclimatic limits and physiological optima, which compete for resources and determine vegetation composition. We used the method outlined by Zhao *et al*.^49^ to adjust a few of the PFT parameters, mainly including the maximum coldest monthly mean temperatures of boreal needle-leaved evergreen forest, boreal needle-leaved summergreen forest and boreal broad-leaved summergreen forest, to fit the characteristics of ecosystems in China.

The original method for calculating AED adds substantially to the uncertainty that is already associated with the climate change signal between GCMs^50^. McVicar *et al*.^51^ advocated that all four primary meteorological variables, i.e., wind speed, atmospheric humidity, radiation, and air temperature, should be considered when assessing trends in AED. However, temperature-based methods, such as the Thornthwaite empirical equation, tend to underestimate AED^52,53^. The Penman–Monteith model recommended by the Food and Agricultural Organization (FAO56-PM model) reference crop evapotranspiration method gave good estimations of spatial and seasonal variability in AED across Great Britain^54^. We therefore modified the original method for calculating AED in the evapotranspiration sub-model of the LPJ^55^ and used the physically-based FAO56-PM model^56^, which had been previously calibrated for the study area^57^. The original fire module in the LPJ assumed only a minimum fuel load for fire spread^58^. The model has been modified to include a maximum fuel load based on the linear relationship between the fuel load and the fire occurrence probability^59^, so that the influence of fuel availability on fire occurrence is better reflected. Li *et al*.^60^ obtained good results when they applied a similar approach to the fire module of CLM-DGVM (Community Land Model with the Dynamic Global Vegetation Model). We expressed the probability of fire based on the available fuel as:

4 $$P=max[0,min(1,\frac{{L}_{{\rm{ag}}}-{L}_{{\rm{low}}}}{{L}_{{\rm{up}}}-{L}_{{\rm{low}}}})]$$

where *L*_ag_ (g C m^−2^) is the fuel load (namely, the above-ground litter), and *L*_low_ (*L*_low_ = 200) and *L*_up_ (*L*_up_ = 1000) are the lower and upper fuel thresholds, respectively. Fire does not occur when *L*_ag_ < *L*_low_, and becomes more likely to occur as the fuel increases when *L*_low_ ≤ *L*_ag_ ≤ *L*_up_. The fuel load no longer limits the fire spread when *L*_ag_ > *L*_low_ when the other conditions are satisfied.

To ensure carbon pools and vegetation coverage were in a state of equilibrium, we ran the LPJ model for an initial period of 1000 years^48^ using the climate data for the reference period from 1981 to 2010 repeatedly. We then ran the simulation from 2011 to 2099 using climate change scenario data and the atmospheric CO_2_ concentration for 2010.

### Climate Projections

Projections of climate change under various scenarios are useful for predicting future changes to ecosystems and their response to global change. We used scenario analysis to assess risk development and the impacts of climate change. Because of the uncertainty associated with GCMs, we simulated forest NPP with multiple GCM projections and used the mean from multiple models to characterize the risk under different emission scenarios. We used five GCMs in this study from the Coupled Model Intercomparison Project Phase 5, including HadGEM2-ES, IPSL-CM5A-LR, GFDL-ESM2M, MIROC-ESMCHEM, and NorESM1-M^61^. The GCM outputs were bias-corrected by the Inter-Sectoral Impact Model Intercomparison Project (ISI-MIP) and downscaled to a spatial resolution of 0.5° ^62,63^. The climate variables were the average, maximum and minimum temperatures, precipitation, surface downwelling shortwave radiation, near-surface wind speed, and relative humidity.

**Figure 2:**
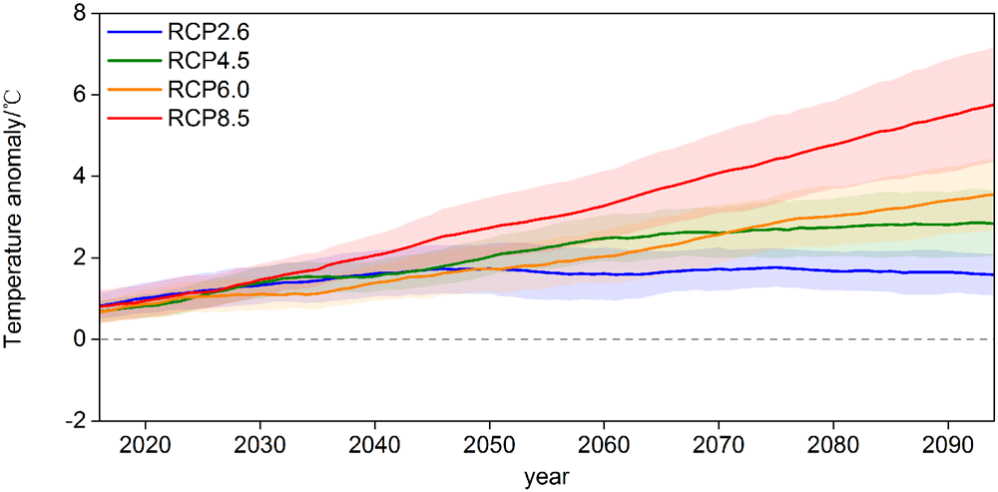
Temperature anomalies in forest areas of China during the 21^st^ century relative to the baseline period (1981–2010). Solid lines indicate the five GCM ensemble means under the RCP scenarios, and the shading indicates one standard deviation of the ensemble mean. The time series were smoothed using a 10-year running mean.

We used four representative concentration pathways, namely RCP2.6, RCP 4.5, RCP 6.0, and RCP8.5 scenarios, which indicated that radiative forcing levels of 2.6, 4.5, 6.0, and 8.5 Wm^−2^ would be reached by 2100, respectively^64^. RCP8.5 was the highest emission scenario with a radiative forcing of around 8.5 Wm^−2^ in 2100, which was equivalent to an atmospheric CO_2_ concentration of about 1370 ppm^64^. By the end of the 21^st^ century, the temperatures in forest areas of China were projected to increase by between 1.68 °C under RCP2.6 and 6.29 °C under RCP8.5 relative to the average temperature in the baseline period of 1981–2010 (Fig. 2).

## Results

### LPJ Model Evaluation

**Figure 3:**
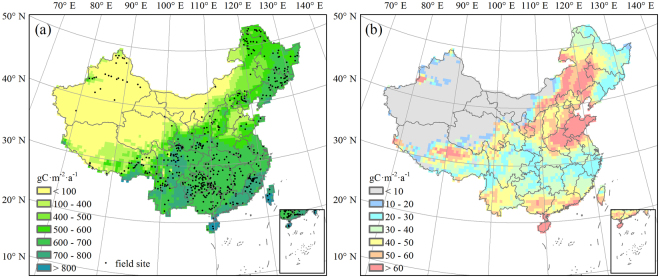
Net primary productivity in China for 1981–2010 and its standard deviation distribution. (**a**) 30-year mean NPP. (**b**) NPP standard deviation. Note that black spots in (**a**) indicate field sites. The field sites data were obtained from the Global Primary Production Data Initiative Products database^70^. The figure was generated using ArcGIS 10.1 software (http://www.esri.com/).

**Figure 4:**
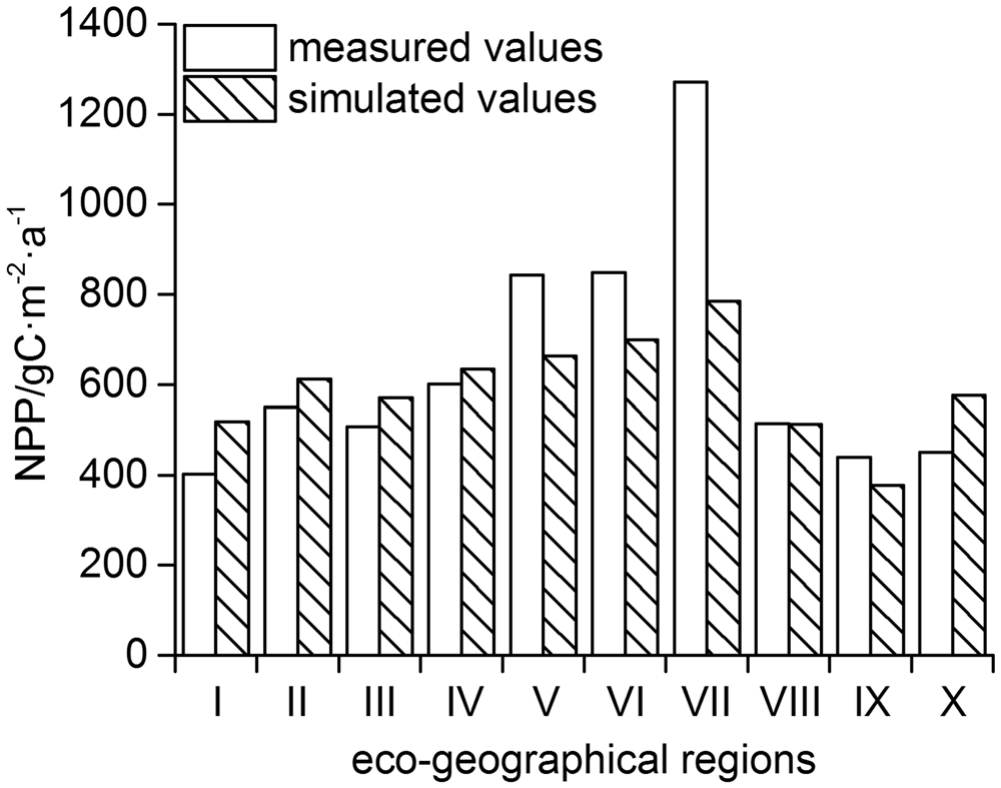
Comparison between simulated and measured net primary productivity in different eco-geographical regions of China. I: Cold temperate humid region; II: Mid-temperate humid/sub-humid region; III: Warm temperate humid/sub-humid region; IV, Northern subtropical humid region; V: Mid-subtropical humid region; VI: Southern subtropical humid region; VII: Tropical humid region; VIII: North semi-arid region; IX: Northwest arid region, and X: Tibetan Plateau region.

The total NPP in China was estimated at 3.61 ± 0.13 Gt C a^−1^ during the baseline period of 1981–2010, which is consistent with the studies of Mao *et al*.^65^, Yuan *et al*.^66^, and Pan *et al*.^67^. The total NPP of forests in China for the past three decades was estimated at 1.40 ± 0.04 Gt C a^−1^, which is close to the results of Zhuang *et al*.^68^ and Ren *et al*.^69^. The 30-year averaged NPP varied spatially and gradually decreased from the southeast to the northwest (Fig. 3a); the vegetation type also varied and transitioned from woodland to grassland. Figure 3b showed the distribution of baseline standard deviation in NPP, which was basically opposite to the pattern of its multi-year mean value and was relatively higher in North China and Hainan Island. The simulated values essentially agreed with the field measurements of NPP for more than 700 sites spread across the different eco-geographical regions in China, published in the Global Primary Production Data Initiative Products database^70^ (Fig. 4). At each field site, a relative error could be estimated from the difference between simulated value and observed value, which was then divided by the observed value and expressed as a percentage. The average relative error over the whole country was 9.94%, which was acceptable and shows that the modified LPJ model gave satisfactory simulations of terrestrial NPP in China, and could be used to predict carbon cycling in ecosystems.

### Temporal change in risk in the future

**Figure 5:**
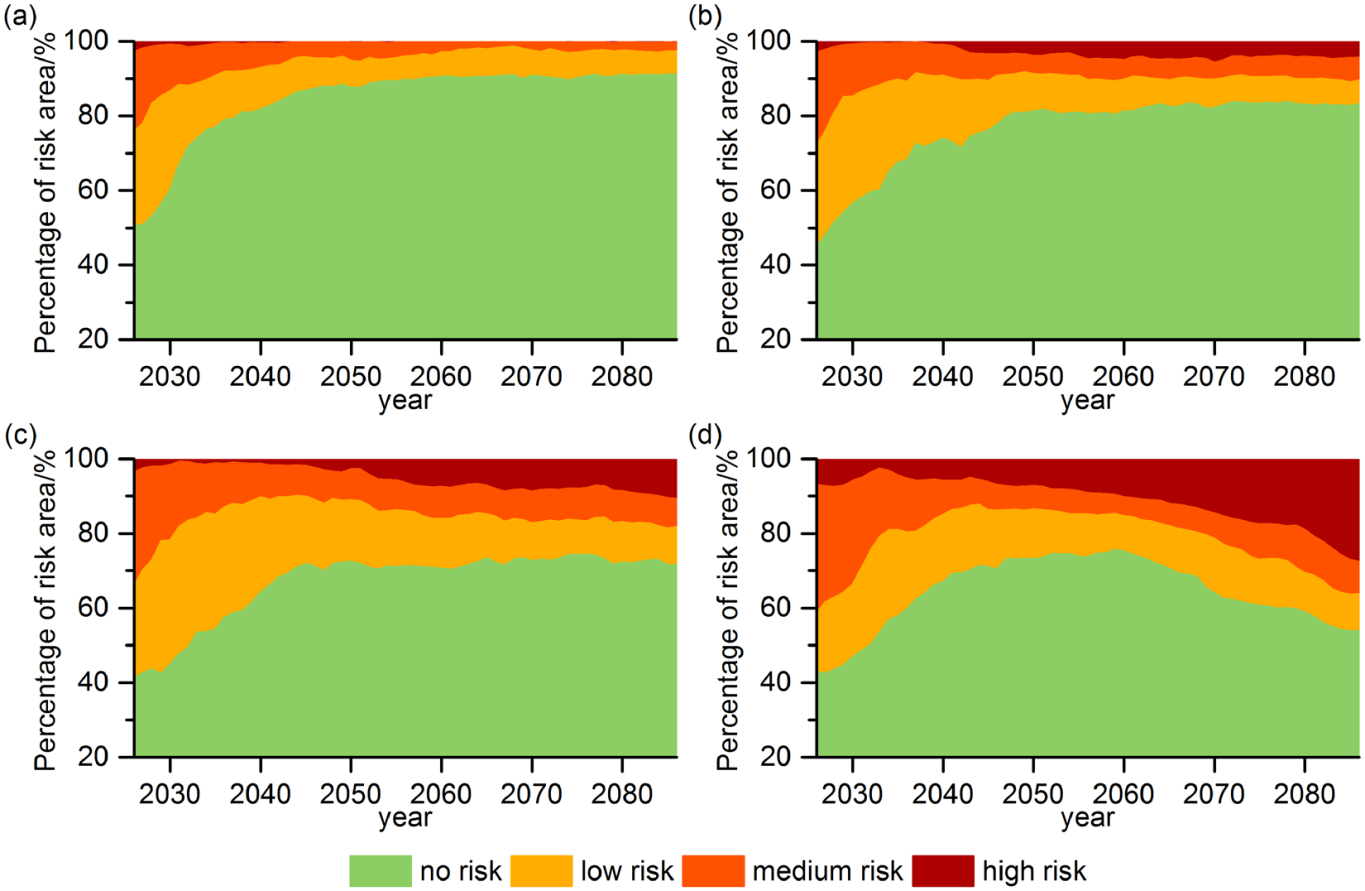
Change of forest area in China at risk during the 21^st^ century under (**a**) RCP2.6, (**b**) RCP4.5, (**c**) RCP6.0, and (**d**) RCP8.5.

Changes in the areas of climate change risk faced by forest systems in China under the four RCPs are shown in Fig. 5. There was a general decrease in the forest area at risk in China in the future, especially under the relatively low emission scenarios. Under RCP2.6, RCP4.5 and RCP6.0, the risk area was predicted to decrease noticeably in the first half of the 21^st^ century and then fluctuate gently in the second half. For the highest emission scenario RCP8.5, the risk area was first predicted to decrease and then to increase from around the 2050s, which was mainly determined by the change in the high-level risk area. The total areas at risk between 2011 and 2099 were predicted to average 15.58%, 23.64%, 33.22% and 36.07% under RCP2.6, RCP4.5, RCP6.0, and RCP8.5, respectively. For the different risk levels, the forest area at medium risk was less than that the area at low risk and did not exceed 10% over most periods, while the area at high risk exhibited significant increasing trends except RCP2.6. Toward the end of the 21^st^ century, the high-risk area was projected to increase at trends of 0.7% (p < 0.01), 1.7% (p < 0.01) and 3.2% (p < 0.01) per decade under RCP4.5, RCP6.0 and RCP8.5, respectively.

### Spatial change of risk in the future

**Figure 6:**
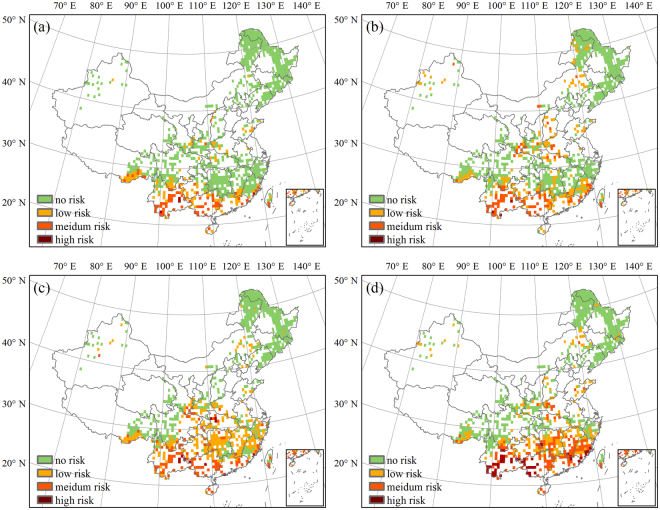
Distribution of forest area at risk in China from 2021 to 2050 under (**a**) RCP2.6, (**b**) RCP4.5, (**c**) RCP6.0, and (**d**) RCP8.5. The figure was generated using ArcGIS 10.1 software (http://www.esri.com/).

To reflect the change in spatial patterns of future risk, two typical periods of 2021–2050 and 2071–2099 were selected in this study, representing mid and long terms. Results from the other periods could be found in Supplementary Figure S1-S4. The risk of climate change to the forest NPP in China is predicted to be aggravated for all scenarios from 2021 to 2050 (Fig. 6). The risk area is predicted to be mainly concentrated in the tropical humid and southern subtropical humid regions under the two relatively low emission scenarios (RCP2.6 and RCP4.5). For the higher emission scenarios (RCP6.0 and RCP8.5), this area is likely to extend northward to the mid-subtropical humid region and the northern subtropical humid region. The total risk areas for the four RCPs covered 21.14%, 31.82%, 42.61%, and 40.24% of the whole forest area (Table 1). Moreover, the low risk area was predicted to account for the largest part of the total risk area, while the high-risk area was predicted to account for the smallest portion. The forest area at low risk was highest under RCP6.0 and was distributed continuously through the areas south of the middle and lower reaches of the Yangtze River. Under RCP8.5, the risks to the forest in the southeast and southwest areas were mainly medium and high. The areas predicted to have medium and high risk under RCP6.0 were predicted to increase significantly from 11.76% and 1.19% to 14.35% and 5.39%, respectively, under RCP8.5.

**Table 1:** Percentage of forest area at risk in China from 2021 to 2050 under different RCPs.

risk level	RCP2.6	RCP4.5	RCP6.0	RCP8.5
low risk	13.05	21.04	29.67	20.50
medium risk	7.66	10.46	11.76	14.35
high risk	0.43	0.32	1.19	5.39
total risk	21.14	31.82	42.61	40.24

**Figure 7:**
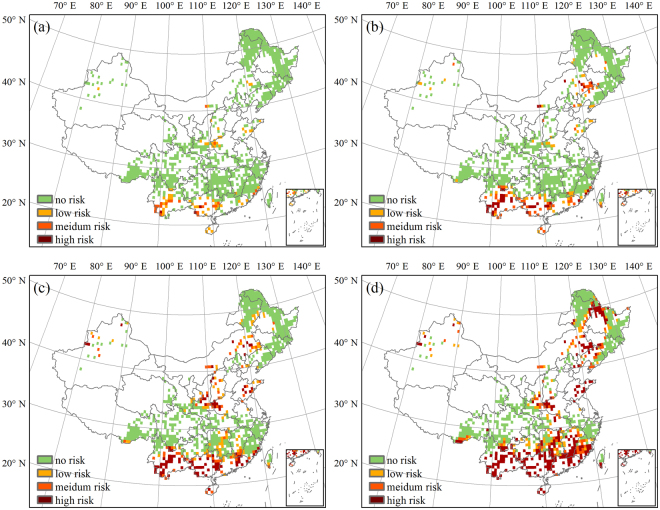
Distribution of forest area at risk in China from 2071 to 2099 under (**a**) RCP2.6, (**b**) RCP4.5, (**c**) RCP6.0, and (**d**) RCP8.5. The figure was generated using ArcGIS 10.1 software (http://www.esri.com/).

The model predicted that the forest NPP risk pattern in China would be noticeably different for the period 2071–2099 (Fig. 7) than for 2021–2050, with the total risk areas accounting for 8.95%, 16.94%, 28.48%, and 46.17% under RCP2.6, RCP4.5, RCP6.0, and RCP8.5, respectively (Table 2). Decreases in the low and medium risk areas mainly explain the sharp decline in the total risk area predicted for the first three RCPs between the periods 2071–2099 and 2021–2050. Some of the low and medium risk areas were predicted to translate into high risk, though this mainly affected areas in southern China. Furthermore, the high-risk area accounted for the largest percentage of the total risk area under RCP6.0 and RCP8.5, while the medium risk area accounted for the smallest portion. The area of forest at risk under RCP8.5 was predicted to be greater during 2071–2099 than 2021–2050 under RCP8.5, mainly because of a significant increase in the high-risk area. This high-risk area was predicted to extend from southern and central China to northeastern China, accounting for 27.62% of the total forest area.

**Table 2:** Percentage of forest area at risk in China from 2071 to 2099 under different RCPs.

risk level	RCP2.6	RCP4.5	RCP6.0	RCP8.5
low risk	6.26	6.47	10.14	9.82
medium risk	2.37	6.04	7.55	8.74
high risk	0.32	4.42	10.79	27.62
total risk	8.95	16.94	28.48	46.17

### Rate of change in risk in response to warming

**Figure 8:**
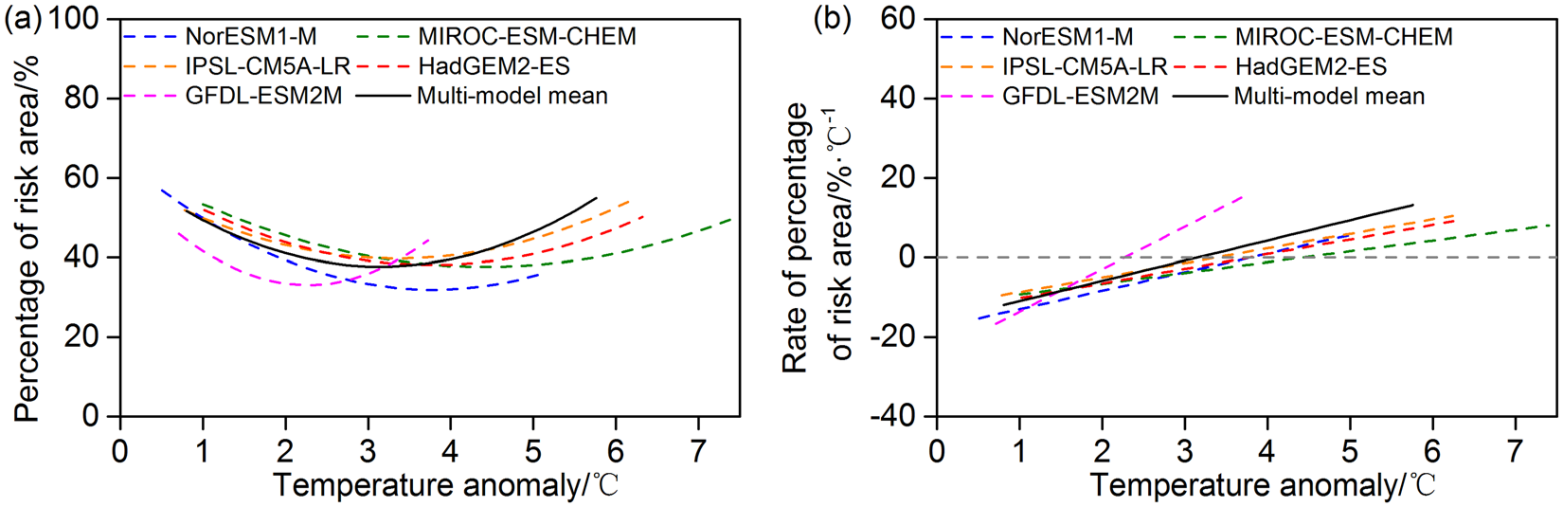
Percentage of forest area affected by risk and its rate of change with temperature in China under RCP8.5. (**a**) Fitted percentage of forest area subject to climate change risk (%) for each model (dashed lines) and the multi-model mean (black solid line). (**b**) Rate of change in the risk area (% °C^−1^) as a function of the temperature anomaly relative to 1981–2010 for each model (dashed lines) and the multi-model mean (black solid line). Before quadratic curve fitting, data were smoothed using a 10-year running mean.

Figure 8a shows changes in the percentage of the area at risk in response to variations in the temperature anomalies predicted by the different GCMs under RCP8.5, processed by quadratic curve fitting. The corresponding rates of change, namely the first derivative of the functions shown in Fig. 8a, are shown in Fig. 8b. When the temperature increased by between 0.80 ± 0.21 °C and 5.76 ± 1.40 °C relative to the baseline (9.76 ± 0.08 °C), the risk area was predicted to first decrease and then increase so that it covered half of the total forest area. The rate of change in the risk area percentage was predicted to first slow down and then accelerate, with the most obvious change predicted by the GFDL-ESM2M. The multi-model mean values show that the NPP risk area made up approximately 38% of the whole forest area in China when the temperature increased by about 3 °C under RCP8.5; for a temperature increase of about 6 °C, the NPP risk area was predicted to extend to almost 55% of the forest area, with a rate of change of 13% °C^−1^.

### Rate of change in risk in response to precipitation change

**Figure 9:**
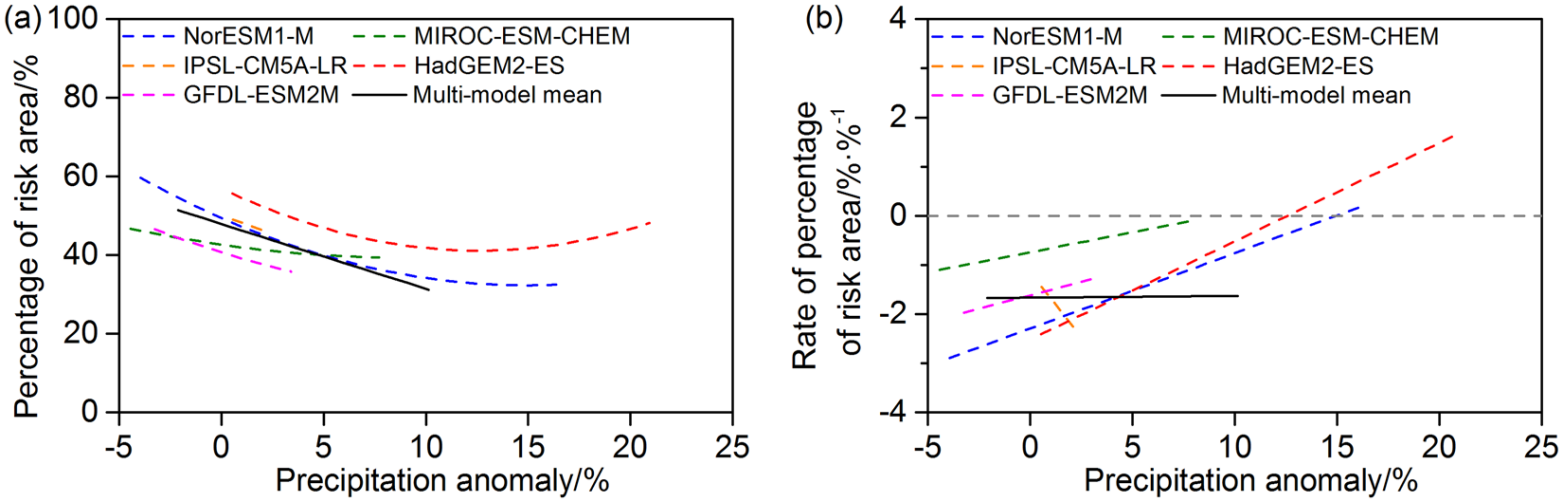
Percentage of forest area affected by risk and its rate of change with precipitation in China under RCP8.5. (**a**) Fitted percentage of forest area subject to climate change risk (%) for each model (dashed lines) and the multi-model mean (black solid line). (**b**) Rate of change in the risk area (% %^−1^) as a function of the precipitation anomaly relative to 1981–2010 for each model (dashed lines) and the multi-model mean (black solid line). Before quadratic curve fitting, data were smoothed using a 10-year running mean.

We further analyzed the rate of change in risk in response to precipitation change. Results showed that the percentage of risk area was projected to decrease in the future, as the precipitation anomaly ranged from −2.11 ± 2.46% to 10.13 ± 8.21% relative to the baseline period (Fig. 9a). For every additional percentage point increase in precipitation, the area at risk tended to decrease by 1.67% of the total forest area (Fig. 9b). In terms of the difference among GCMs, IPSL-CM5A-LR showed a small range of precipitation change and a quick drop of NPP risk area; the area at risk was projected to turn from decreasing to increasing, when the precipitation increased by about 13% in the case of HadGEM2-ES.

### Driving factors

**Figure 10:**
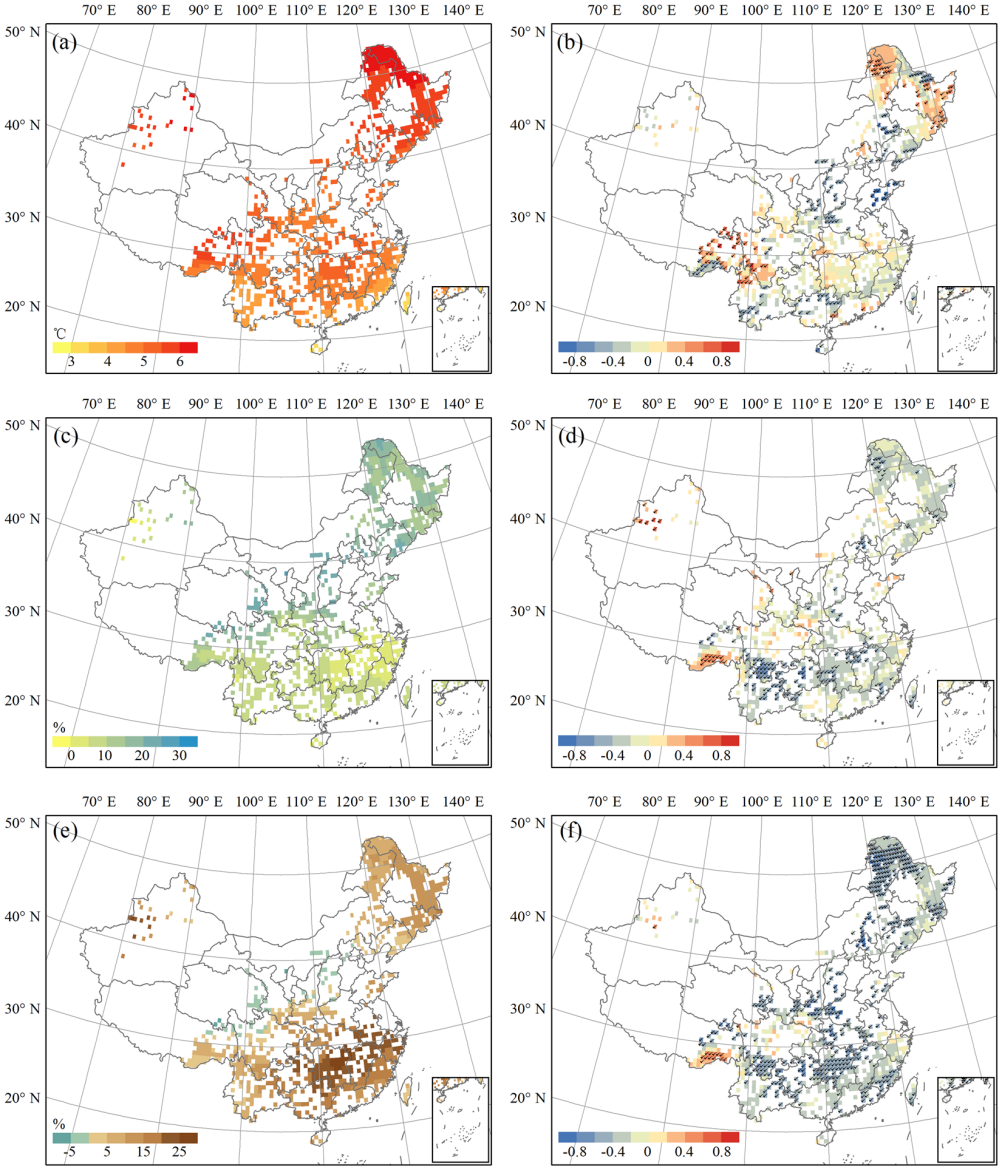
Anomalies of climatic factors during 2071–2099 relative to 1981–2010 under RCP8.5 and their partial correlation coefficients with forest NPP in China. (**a**,**b**) temperature. (**c**,**d**) precipitation. (**e**,**f**) aridity index. Note that black checkmarks indicate statistical significance (p < 0.05). The figure was generated using ArcGIS 10.1 software (http://www.esri.com/).

We attributed the IAV of NPP to different climatic factors by calculating the partial correlation coefficients between NPP and precipitation (PRE), temperature (TEM), and aridity index (AED/PRE) for the period 2071–2099 under RCP8.5 (Fig. 10). There were significant positive correlations between temperature and forest NPP, mainly in northeastern China and in the eastern part of the Tibetan Plateau, which occupied 9.60% of the total forest area. Significant negative correlations between precipitation and forest NPP accounted for a larger proportion (15.53%) and were mainly limited to southern China. Substantial temperature rise was projected to occur in Northeast China, whereas the relatively slight increase of precipitation seemed to be distributed in Southeast China. The aridity index tended to increase obviously for these areas, implying potential droughts in the long term under high emission scenario. The aridity index and NPP were significantly and negatively correlated for nearly half of the forests (49.84%), ranging from northeastern China to southern China, indicating that increased dryness would limit forest growth in these regions. Therefore, of the three variables, the projected aridity index seemed to have a greater impact on the forest NPP, both in its degree and extent, which means that risks of a decrease in the forest NPP were mainly because of dryness.

## Discussion

The pattern predicted in this study is spatially consistent with other recent findings. For example, Gang *et al*.^71^ suggested that the terrestrial NPP would increase in the northern mid and high latitudes where warming would favor tree growth and expansion by the end of the 21^st^ century, especially for temperate and boreal forests. Using the RCP4.5 scenario in the Integrated Biosphere Simulator, Yuan *et al*.^72^ found that the NPP of deciduous broad-leaved forest in the warm temperate zone in China would increase, while that of subtropical evergreen broad-leaved forests in southern China would decrease, and would be vulnerable from 2016 to 2050.

We found that the total area of forest in China at risk from climate change would first decrease and then increase as the warming accelerated under RCP8.5. The future projections of forest risk to climate change indicate that climate warming may be beneficial to vegetation growth to a minor extent, but when the climate change exceeds a certain threshold, the impacts could be negative. While increases in temperature could enhance plant photosynthesis, they could also cause a water vapor pressure deficit. The leaf stomata would then close to prevent water loss and increase water use efficiency^73^. Several previous studies have reported that, without considering the CO_2_ fertilization effect, rapid temperature increases and increased frequency of drought events could cause a decrease in the NPP in tropical and subtropical forests, and even in the global terrestrial NPP, under relatively high emission scenarios^71,74^. Because of warming and changes in rainfall patterns, the decrease in the available soil water has slowed down the increase in the forest NPP in southern China over the past three decades^75^. From their analysis of 32 years of data from forest observation plots, Zhou *et al*.^76^ suggested that subtropical forests in China were threatened by their lack of resilience to long-term climate change manifested by rising temperatures and increased occurrence of soil drying. Also, studies of the impact of climate change on aridity during the 21^st^ century have predicted increased aridity over most tropical and mid-latitude land regions^37^, and, in particular, over most of Africa, the Americas, Australia, Southeast Asia, and the Mediterranean region^77,78^. This indicates that more intense droughts would limit future forest growth in low latitude regions. Climate change alone may lead to less overall tree coverage in the Tropics, while the competing effects of CO_2_ fertilization and climate change, along with the uncertainty of projected precipitation changes in the Tropics, mean that there is a large degree of uncertainty associated with projected future changes in vegetation^79^.

Moreover, shifts in forest disturbances such as wind, pests, and fire may adversely affect forest productivity under future climate change. The productivity of and carbon storage in Europe’s forests is likely to decrease as climate change and forest disturbances intensify^31,80^, though both increasing and decreasing trends have been found in the growth and productivity across Europe^81–83^. For example, the growth of European beech was observed to have declined because of droughts over the past 20th century^80^, and its NPP and water-use efficiency was predicted to reduce under future climate conditions (A1B scenario) due to aggravated water shortage and droughts^84^. As the risks from forest fire are predicted to become increasingly serious in China, mainly because of an increase in fire weather in central and southeastern China^85^, measures should be implemented to reduce the negative impacts of fire disturbance on forest productivity. The sensitivity of NPP to climate change is also the key to understanding how risk develops and evolves. Piao *et al*.^21^ found that the inter-annual correlation between terrestrial productivity and temperature decreased in temperate regions because of an increase in drought over the past decade, while Heyder *et al*.^16^ reported that strong warming could amplify the sensitivity to declining precipitation in temperate and tropical ecosystems.

There are uncertainties in risk forecasts in vegetation productivity from several sources, including emission scenarios, climate models, and ecological models. For instance, due to the large discrepancy in future projections of precipitation from global to regional scale^86,87^, the difference in precipitation patterns and extreme events across CMIP5 models could be a vital source of uncertainty for terrestrial carbon flux and its impact from climate change^88^. As for China, the cross-model variability of future NPP was reported to be significantly contributed by the simulated precipitation on the local scale, especially in northwestern area^89^. From their assessment, Sitch *et al*.^90^ found that the responses of five DGVMs to climate change varied more widely than their responses to changes in CO_2_ concentrations. Nishina *et al*.^91^ considered that, if the uncertainty in the ISI-MIP results were to be reduced, the simulation capacity of vegetation models would need to improve. When estimating forest biomass and productivity, accurate descriptions and determinations of allometry and allometric scaling parameters, respectively, are important^92^. We did not consider the direct effect of CO_2_ fertilization in our study even though it is an important influence on changes in vegetation NPP. The observed increase in photosynthetic water-use efficiency in temperate and boreal forests of the Northern Hemisphere over the past two decades has been closely associated with elevated atmospheric CO_2_ concentrations^93^. Water stress or decreased carbon gains from autotrophic respiration may result in decreased vegetation productivity and loss of forest cover when there are shortages of CO_2_^94^. There was a considerable difference between the simulated and observed data in the forest NPP in the tropical humid region for the baseline period, which probably reflects the fact that there were only seven site records from this area, thereby causing high uncertainty. To measure this kind of uncertainty, the time series of observed NPP from 1981 to 2010 are needed to compute the standard deviation and to further calculate the risk. Nevertheless, due to the lack of time series data in the observed NPP across China^70,95–97^, we are unable to quantify the uncertainty resulting from the difference between simulations and observations. Further, different ways of assessing risk may also create uncertainties. For example, baseline values may differ depending on whether they are derived from the global average or regional average, and variable values may exceed natural variability when they are beyond the mean plus or minus several standard deviations^5,16,47^. All these factors can produce different risk assessment results. van Oijen^98,99^ defined risk as the product of probability and vulnerability, and used the difference in NPP between hazardous condition and normal climate to indicate ecosystem vulnerability. Although the method is explicit in mathematics and easy to operate, it seems more suitable to the risk induced by single hazard factor and the distinction between hazardous and non-hazardous conditions mainly depended on the subjective experience. In addition to the impact of climate change, human activities like forest management measures may also drive changes in ecosystem^100^. It deserves more research on how to isolate the climate change risk from its complicated interactions with human factors.

## Conclusion

In this study, we modified the AED and fire modules of the LPJ model and investigated spatial and temporal features of climate change risk faced by forest in China in the 21th century under different RCPs. The risk rating predicted in this study from the relationships between decreases in NPP and baseline variability (indicated by the standard deviation) highlighted the adverse impact of future climate change on vegetation productivity. The area of forest NPP at risk in China showed a general tendency to decrease from 2011 to 2099 relative to the baseline period of 1981–2010, under RCP2.6, RCP4.5 and RCP6.0. High-level risk area would increase especially in RCP4.5 (0.7% per decade, p < 0.01) and RCP6.0 (1.7% per decade, p < 0.01).

The risk of climate change to forest in China is likely to be relatively obvious under RCP8.5 compared with low emission scenarios especially in the long term. In response to future climate change, the total risk area is predicted to first decrease and then increase after the middle of 21^st^ century. The percentage area at high risk was predicted to increase from 5.39% (2021–2050) to 27.62% (2071–2099) with a trend of 3.2% per decade (p < 0.01). The forest vegetation growth would probably be weakened as the degree of warming increased under RCP8.5.

Spatial distributions show that climate change risk to forests was projected to be concentrated in south China. The risk of future climate change to forest in China is predicted to be mainly distributed in the low latitude southern subtropical humid and tropical humid regions where there were intensified dryness and where the declines in productivity superimposed by natural hazards such as droughts and floods may bring huge losses to the local economy.

## Electronic supplementary material

Supplementary Information
