# Supplementary material for: Climate change risk to forests in China associated with warming
Source: Sci Rep. 2018 Jan 11;8:493. doi: 10.1038/s41598-017-18798-6 (PMC5765107; doi:10.1038/s41598-017-18798-6)
Supplement: Supplementary file 1 — Supplementary Information [file 41598_2017_18798_MOESM1_ESM.pdf]

Climate change risk to forests in China associated with warming

Yunhe Yin[1,*], Danyang Ma [1,2], Shaohong Wu [1,2]

[1] Key Laboratory of Land Surface Pattern and Simulation, Institute of Geographic Sciences and Natural Resources Research, Chinese Academy of Sciences, 11A, Datun Road, Chaoyang District, Beijing 100101, China

[2] University of Chinese Academy of Sciences, 19A Yuquan Road, Shijingshan District, Beijing 100049, China

[*] Corresponding author

E-mail: yinyh@igsnrr.ac.cn

Contents of this file

Figure S1

Figure S2

Figure S3

Figure S4

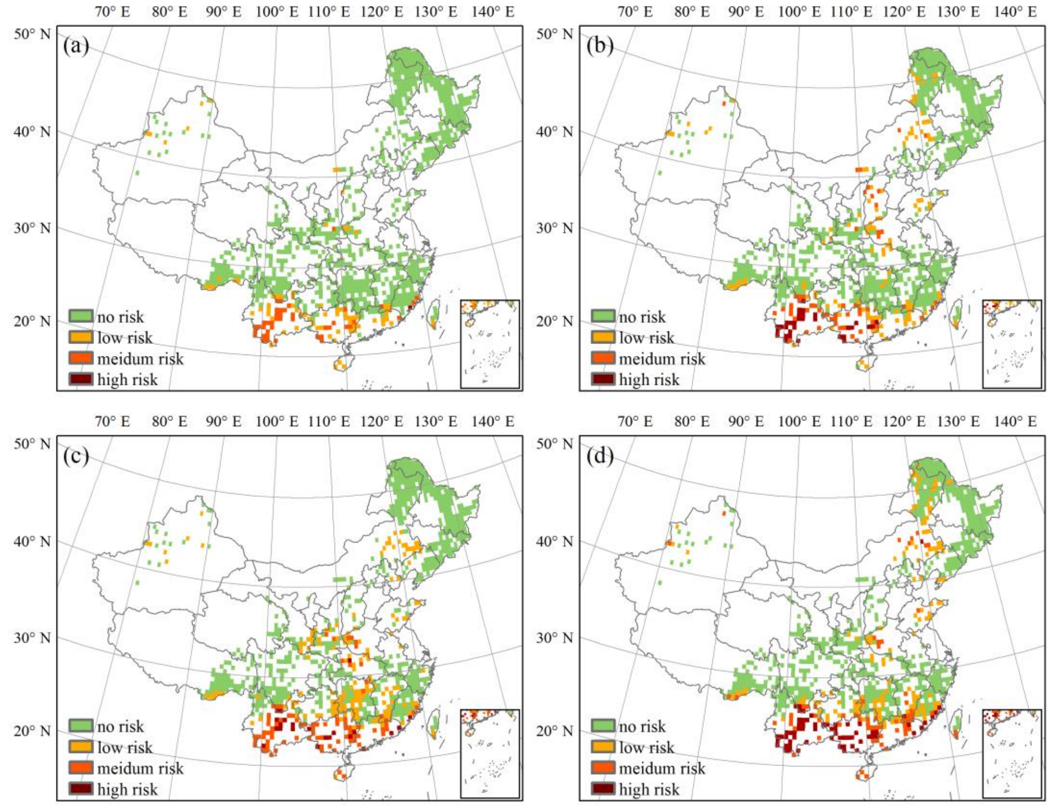

Figure S1. Distribution of forest area at risk in China from 2031 to 2060 under (a) RCP2.6, (b) RCP4.5, (c) RCP6.0, and (d) RCP8.5. The figure was generated using ArcGIS 10.1 software (http://www.esri.com/).

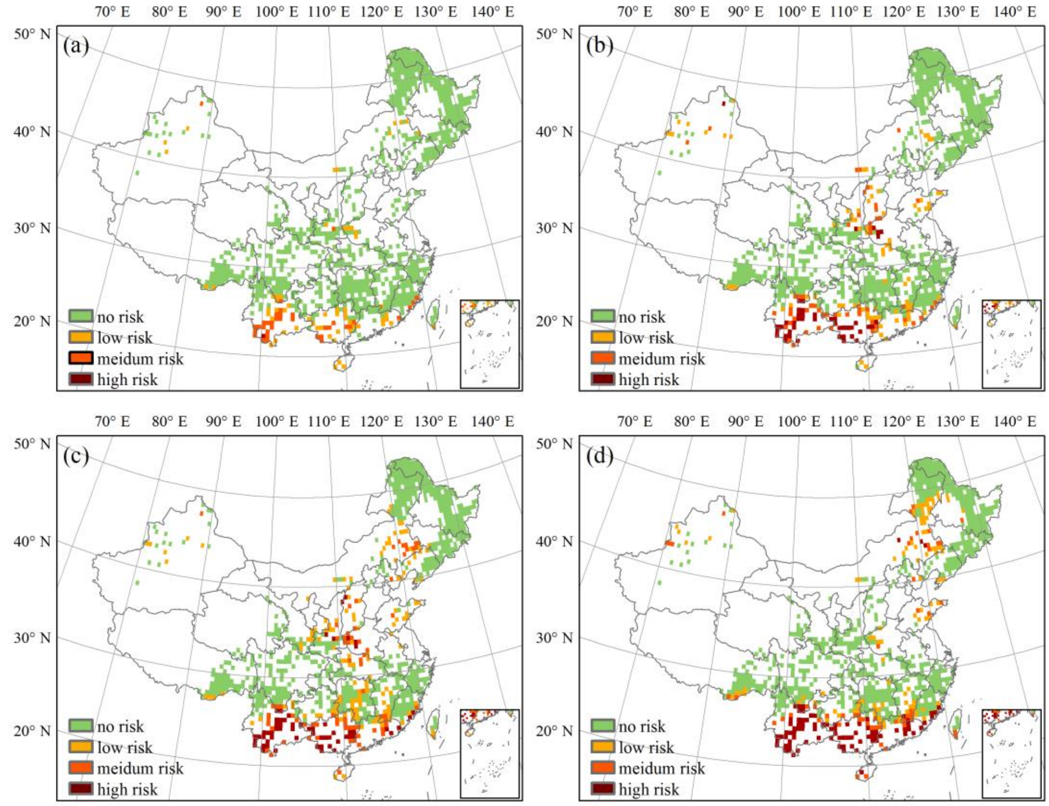

Figure S2. Distribution of forest area at risk in China from 2041 to 2070 under (a) RCP2.6, (b) RCP4.5, (c) RCP6.0, and (d) RCP8.5. The figure was generated using ArcGIS 10.1 software (http://www.esri.com/).

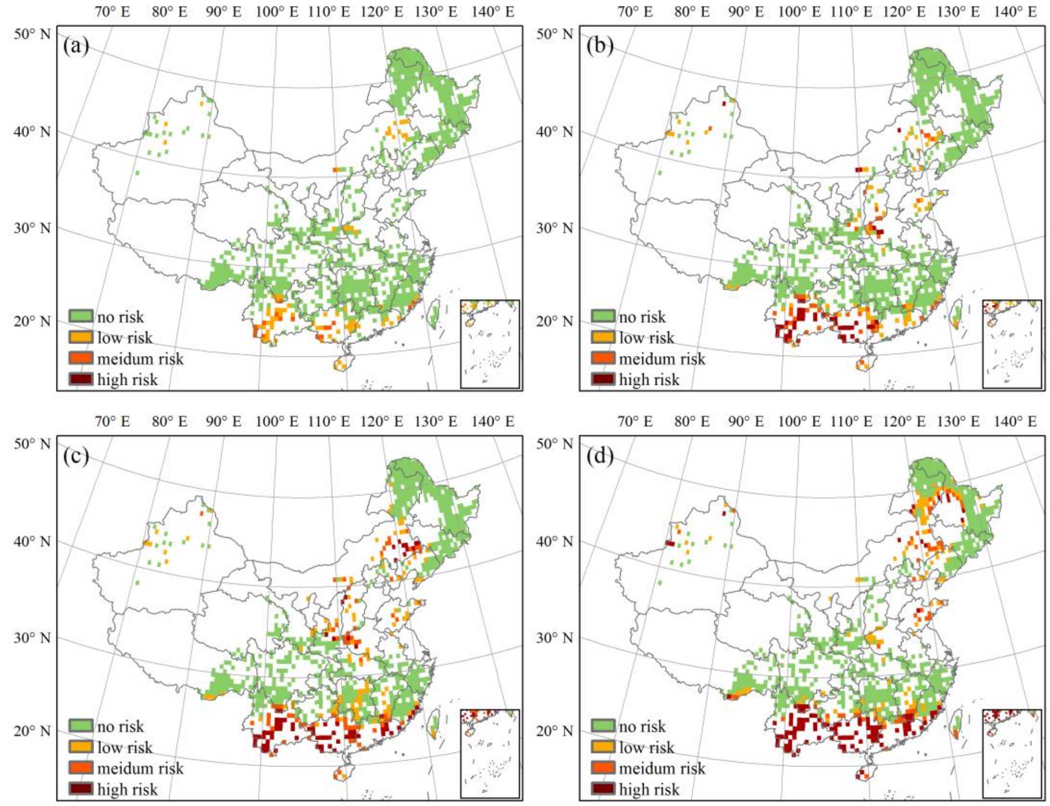

Figure S3. Distribution of forest area at risk in China from 2051 to 2080 under (a) RCP2.6, (b) RCP4.5, (c) RCP6.0, and (d) RCP8.5. The figure was generated using ArcGIS 10.1 software (http://www.esri.com/).

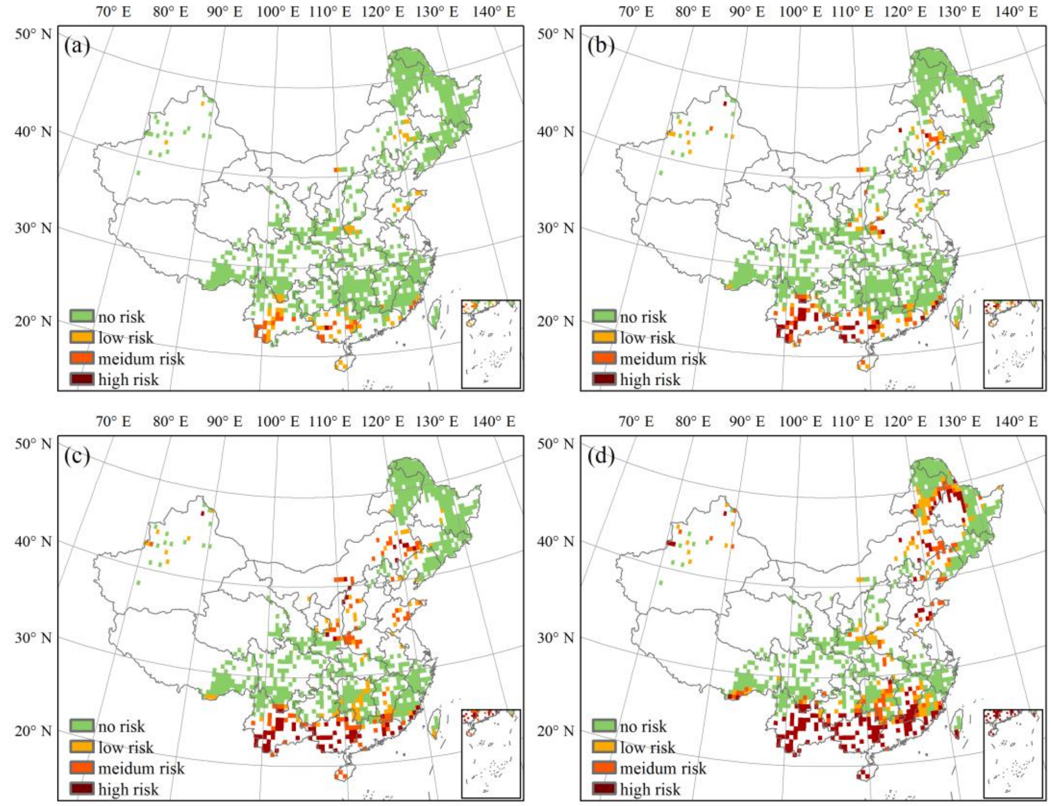

Figure S4. Distribution of forest area at risk in China from 2061 to 2090 under (a) RCP2.6, (b) RCP4.5, (c) RCP6.0, and (d) RCP8.5. The figure was generated using ArcGIS 10.1 software (http://www.esri.com/).
